# Pharmacological Role of Anions (Sulphate, Nitrate, Oxalate and Acetate) on the Antibacterial Activity of Cobalt(II), Copper(II) and Nickel(II) Complexes With Nicotinoylhydrazine-Derived ONO, NNO and SNO Ligands

**DOI:** 10.1155/MBD.1996.211

**Published:** 1996

**Authors:** Zahid Hussain Chohan, Abdur Rauf

**Affiliations:** 1 Department of Chemistry Islamia University, Bahawalpur, and Bahauddin Zakariya University Multan Pakistan

## Abstract

Mixed ligands biologically active complexes of cobalt(II), copper(II) and nickel(II) with nicotinoylhydrazine-derived ONO, NNO and SNO donor schiff-base ligands having the same metal ion but different anions such as sulphate, nitrate, oxalate and acetate have been synthesised and characterised on the basis of their physical, analytical and spectral data. In order to evaluate the role of anions on their bioability, these ligands and their synthesised metal complexes with various anions have been screened against bacterial species such as *Escherichia coli, Pseudomonas aeruginosa* and *Staphylococcus aureus* and the title studies have proved a definative role of anions in increasing the biological activity


### PHARMACOLOGICAL ROLE OF ANIONS (SULPHATE, NITRATE, OXALATE AND ACETATE) ON THE ANTIBACTERIAL ACTIVITY OF COBALT(II), COPPER(II) AND NICKEL(II) COMPLEXES WITH NICOTINOYLHYDRAZINE- DERIVED ONO, NNO AND SNO LIGANDS

Zahid Hussain Chohan* and Abdur Rauf

Department of Chemistry, Islamia University, Bahawalpur, and
Bahauddin Zakariya University, Multan, Pakistan

### ABSTRACT

Mixed ligands biologically active complexes of cobalt(II), copper(II) and nickel(II) with nicotinoylhydrazine-derived ONO, NNO and SNO donor Schiff-base ligands having the same metal ion but different anions such as sulphate, nitrate, oxalate and acetate have been synthesised and characterised on the basis of their physical, analytical and spectral data. In order to evaluate the role of anions on their bioability, these ligands and their synthesised metal complexes with various anions have been screened against bacterial species such as *Escherichia coli*, *Pseudomonas aeruginosa* and *Staphylococcus aureus* and the title studies have proved a definative role of anions in increasing the biological activity

### INTRODUCTION

The relationship[1-5] between metal ions and biological systems is currently obvious and hence, is a subject of great interest for researchers. A bulk of literature[6-13] has drawn attention to this fact of the most significant nature and use of metal ions for life and their role in different biochemical processes. For example, functioning of many enzymes is metal ion dependent[14,15]. The metal ion may induce by coordination a specific "Lock geometry" of the apoprotein metal binding site so that only certain substances are able to become attached to the framework formed by the result of such interaction.

The increasing interest in this area, also attracted us to investigate and enlighten the biological role[16-23] of metal ions in chelation and subsequently increasing the pharmacological properties. Paralleling the same concepts, we have furthermore investigated for the first time the possible role[24-26] of anions (counter part of metal ions) in biological systems and in extension to the same, now, wish to report this role of anions (sulphate, nitrate, oxalate and acetate) on biologically active metal(II) complexes of nicotinoylhydrazine-derived Schiff-base ligands reported[27] earlier by us. The present studies principally consist of the formation of various metal(II) complexes having the same metal ion but different anions represented by a general formula $[M(L)_2 X_2]$ where M = Co(II), Cu(II) and Ni(II), L = $L_1$, $L_2$ and $L_3$ (Fig 1) and X = $SO_4$, $NO_3$, $C_2O_4$ and $CH_3CO_2$, and then investigating the role of anions on the antibacterial activity against bacterial species *Escherichia coli*, *Pseudomonas aeruginosa* and *Staphylococcus aureus*

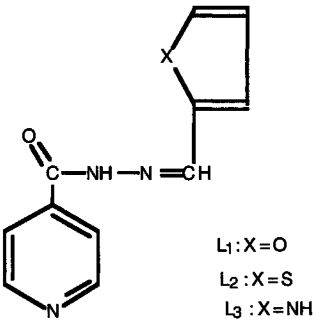

Figure 1 Structure of the ligand

* Present address for correspondence: Department of Chemistry, Meston Walk, University of Aberdeen, Old Aberdeen AB9 2UE, Scotland (U.K.)

### EXPERIMENTAL

### Material and Methods

All solvents and chemicals used were of AR grade. All metals were used as their sulphate, nitrate, oxalate and acetate salts in the preparation of complexes. Conductance and magnetic measurements were made using a YSI model-32 conductivity bridge and Gouy balance, respectively.

Infrared spectra (nujol) were recorded in a A-10 Hitachi spectrophotometer. $^1H$ NMR spectra in DMSO $d_6$ were obtained on a R10-Perkin Elmer spectrometer. Electronic spectra in DMF were studied on a Hitachi double-beam U-2000 model spectrophotometer using glass cells of 1 cm thickness. Elemental analysis of C, H, N were determined on a Coleman automatic analyser. All melting points were taken on a Gallenkamp melting point apparatus and are uncorrected. Antibacterial studies were carried out with the help of the Microbiology Laboratory, Department of Pathology, Qaid-e-Azam Medical College, Bahawalpur.

### Preparation of Ligands

Nicotinoylhydrazine-derived ligands ($L_1$, $L_2$ and $L_3$) (Figure 1) were prepared and characterised (Table 1) according to the method reported[27] earlier by us.

### Preparation of Metal Complexes

To a hot *n*-butanol solution (25 mL) of the ligand (0.004 mol) was added an ethanolic solution (15 mL) of the metal salt (0.002 mol). The mixture was refluxed for 2 h.The resulting mixture was cooled, filtered and reduced to nearly a half of its volume (20 mL) by an evaporator. The concentrated solution so obtained was left overnight at room temperature which resulted in the formation of a solid product. The solid product thus formed was filtered, washed with *n*-butanol (2x10 mL) and dried. Crystallisation of the crude product in aqueous *n*-butanol (50 %) gave the desired metal complexes **1-36** (Table 2).

### Antibacterial Studies

The synthesised metal complexes and ligands were screened for their antibacterial activity against bacterial species *Escherichia coli (a)*, *Pseudomonas aeruginosa (b)* and *Staphylococcus aureus (c)*. The paper disc diffusion method deviced and reported earlier[17,20] by us was used for the determination of antibacterial activity.

### Preparation of Discs

Metal complexes/ligands (30 μg) in DMF (0.01 mL) were applied on a disc prepared from blotting paper (3 mm diameter) with the help of a micropipette. The discs were incubated at 37°C for 48 h and then applied on specific bacteria grown agar plates.

### Preparation of Agar Plate

Minimal agar was used for the growth of specific bacterial species. For this purpose different media were used specifically according to the proposed method[28] of Merck.

### Method of Inoculation

A platinum wire loop was used for inoculation which was first made red hot in a flame, cooled in air and then used for the application of specific bacterial strains. The preculture was prepared in 2 mL of nutrient broth by selecting a suitable colony of bacteria and then transfering it to the nutrient broth. It was incubated for 2 h at 37°C. Then 500 μL of the culture was spread on the specific agar plate and incubated for 24 h at 37°C.

### Application of Discs

A sterilised forecep was used for the application of disc on already incubated agar plates. When the disc was applied, it was then incubated at 37°C for 2 h. The diameter of the zone of inhibition was measured.

### RESULTS AND DISCUSSION

The structure of the title ligands (Table 1) was established as reported[27] earlier and structure of their metal complexes synthesised in the present studies were established with the help of their physical, spectral and analytical data (Table 2). All the metal complexes were prepared by a stoichiometric reaction of metal(II) salt having different anions and the respective ligands in a molar ratio M:L = 1:2 (Fig 2). All complexes formed are air and moisture stable solids.

$$2L + MX \rightarrow [M(L)_2]X$$

$L = L_1, L_2$ and $L_3$

$M$ = Co(II), Cu(II) and Ni(II)

$X = SO_4, NO_3, C_2O_4$ and $CH_3CO_2$

They are soluble in DMSO, DMF and water and insoluble in other solvents. The conductivity of these complexes (12-20 ohm$^{-1}$ cm$^{2}$ mol$^{-1}$) in DMF shows all of them to be non-electrolytes[28,29]. Their solubility, crystalline nature and melting behaviour suggest that they are all non-polymeric.

**Table 1** Physical, Spectral and Analytical Data of Ligands

| Ligand/ Mol.Form | Yield (%) | M.P. ($^0$C) | IR (cm$^{-1}$) | $^1$H-NMR (ppm) | Cal(%)(Found) C | H | N |
|---|---|---|---|---|---|---|---|
| $L_1$ $C_{10}H_9N_3$ | 46 | 157 | 3280,2810,2575, 2024,1670,1620, 1550,1375,1112, 1020,970 | 6.18(2H,DD,H-3,4),6.82(1H,d, H-6),7.85(3H,m,H-1,4,5),7.28 (2H,dd,H-2,3),8.85(1H,s,NH, Exchangeable) | 59.11 (59.13) | 4.43 (4.41) | 20.67 (20.64) |
| $L_2$ $C_{10}H_9N_3S$ | 50 | 149 | 3285,2810,2570, 2025,1670,1620, 1555,1375,1115, 1025,970 | 6.2(2H,dd,H-3,4),6.9(1H,d,H-6) 7.85(3H,m,H-1,4,5),7.28(2H, dd,H-2,3),8.9(1H,s,NH, Exchangeable) | 59.11 (59.08) | 4.43 (4.48) | 20.67 (20.6) |
| $L_3$ $C_{10}H_{10}N_4$ | 50 | 135 | 3280,2815,2570, 2025,1670,1620, 1550,1375,1115, 1020,1010,970, | 6.2(2H,dd,H-3,4),6.85(1H,d,H-6) 7.9(3H,m,H-1,4,5),7.25(2H,dd, H-2,3),8.95(2H,br s,NH, Exchangeable) | 64.53 (64.59) | 5.37 (5.38) | 30.09 (30.15) |

### Infrared Spectra

The prominent IR frequencies of the complexes are listed in Table 2. The scrutiny of important bands exhibited by the ligands and their complexes show that a band at 3280 cm$^{-1}$ in the spectra of free ligands assigned to ν(NH) remains unchanged in the spectra of their metal complexes thus indicating that ligands are not coordinated through -NH. However, a strong band in the spectra of ligands at 1670 cm$^{-1}$ is due to ν(C=O) stretching distinctly shifted towards lower frequency by 30-40 cm$^{-1}$ indicative of the participation of this carbonyl group in coordination. IR spectra of all ligands exhibit a band at 1620 cm$^{-1}$ due to azomethine moiety which also shifted towards lower frequency by 5-10 cm$^{-1}$ invariably in all the complexes suggesting respectively, that the ligands are coordinated to the metal ion via C = 3DN. In the far infrared region three bands around 375-382 cm$^{-1}$, 445-450 cm$^{-1}$ and 510-525 cm$^{-1}$ are observed for all the complexes, which are not found in the spectrum of the free ligand. These are assigned to ν(M-S), ν(M-O) and ν(M-N) vibrations respectively, providing a conclusive evidence[30-32] for the participation of the heteroatom of the heterocyclic ring system in coordination.

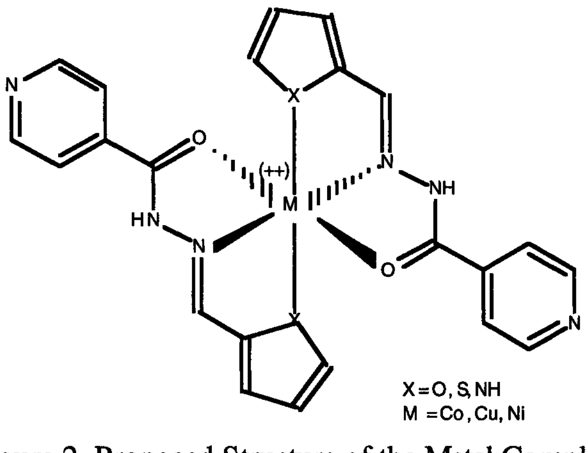

Figure 2 Proposed Structure of the Metal Complex

### Electronic Spectra and Magnetic Moments

The nature of the ligand field around the metal ion and the geometry of the complexes have been deduced from the electronic spectral data and magnetic moment values given in Table 2. Co(II) complexes show three absorption bands $4T_{1g}(F) \rightarrow 4T_{2g}(F)(V_1)$ at 8140-10215 cm$^{-1}$ $4T_{1g}(F) \rightarrow 4A_{2g}(F)(V_2)$ at 17545-18770 cm$^{-1}$ and $4T_{1g}(F) \rightarrow 4T_{1g}(P)(V_3)$ at 21980-23450 cm$^{-1}$ in solution having assigned[33,34] to octahedral geometry Similarly, Ni(II) complexes show three absorption bands between 10265-11530, 18285-19050 and 26670-29955 $cm^{-1}$ corresponding to $3A_{2g} \rightarrow 3T_{2g}(V_1)$, $3A_{2g} \rightarrow 3T_{1g}(F)(V_2)$ and $3A_{2g} \rightarrow 3T_{1g}(V_3)$ transitions respectively, characteristic for their octahedral stereochemistry[35]. Cu(II) complexes exhibit three bands in the regions 29415-30650, 22255-23585 and 16225-18660 $cm^{-1}$. The lower energy band may be assigned as the 10 Dq band for a distorted octahedral geometry [36] corresponding to the transitions $2E_g \rightarrow 2T_{2g}$. The band at 22255-23585 $cm^{-1}$ may be due to intra-ligand charge transfer.

**Table 2.** Physical, Spectral and Analytical Data of Metal Complexes

| No | Mol.Formula | yield (%) | M.P. (°C) | B.M ($\mu_{eff}$) | IR ($cm^{-1}$) | $\lambda_{max}$ ($cm^{-1}$) | Cal(%)(Found) C | H | N |
|---|---|---|---|---|---|---|---|---|---|
| 1 | $[Co(L_1)_2(SO_4)]$ $C_{22}H_{18}CoN_6O_8S$ | 51 | 220-222 | 4.8 | 3280(NH),1665(C=O) 1612(C=N),445(M-O), 510(M-N). | 8140,18770, 20450. | 45.15 (45.02) | 3.07 (3.39) | 14.35 (14.68) |
| 2 | $[Co(L_1)_2(NO_3)_2]$ $C_{22}H_{18}CoN_8O_{10}$ | 52 | 206-208 | 4.91 | 3280(NH),1652(C=O), 1615(C=N),442(M-O), 525(M-N). | 9115,17545, 21950. | 43.09 (42.88) | 2.93 (3.16) | 18.26 (18.54) |
| 3 | $[Co(L_1)_2(C_2O_4)]$ $C_{24}H_{22}CoN_6O_8$ | 50 | 217-219 | 4.97 | 3280(NH),1658(C=O), 1610(C=O),445(M-O), 510(M-N). | 8280,17670, 22220. | 49.59 (49.21) | 3.78 (3.22) | 14.45 (14.76) |
| 4 | $[Co(L_1)_2(CH_3CO_2)_2]$ $C_{26}H_{24}CoN_6O_8$ | 53 | 202-204 | 5.22 | 3280(NH),1643(C=O), 1610(C=N),450(M-O), 510(M-N). | 10215,18590, 23450. | 51.42 (51.86) | 3.95 (4.22) | 13.83 (13.72) |
| 5 | $[Cu(L_1)_2(SO_4)]$ $C_{22}H_{18}CuN_6O_8S$ | 49 | 198-200 | 1.52 | 3280(NH),1658(C=O), 1614(C=N),445(M-O), 515(M-N). | 16250,22255, 30650. | 44.79 (44.38) | 3.05 (2.89) | 14.24 (14.33) |
| 6 | $[Cu(L_1)_2(NO_3)_2]$ $C_{22}H_{18}CuN_8O_{10}$ | 50 | 195-197 | 1.57 | 3280(NH),1661(C=O), 1615(C=N),445(M-O), 515(M-N). | 17180,23585, 29415. | 42.77 (42.81) | 2.91 (2.76) | 18 13 (18.01) |
| 7 | $[Cu(L_1)_2(C_2O_4)]$ $C_{24}H_{22}CuN_6O_8$ | 48 | 197-199 | 1.55 | 3280(NH),1665(C=O), 1615(C=N),450(M-O), 525(M-N). | 18215,23190, 30575. | 49.20 (49.67) | 3.75 (3.77) | 14.33 (14.28) |
| 8 | $[Cu(L_1)_2(CH_3CO_2)_2]$ $C_{26}H_{24}CuN_6O_8$ | 51 | 200-202 | 1.58 | 3280(NH),1640(C=O), 1612(C=N),510(M-N), 445(M-O). | 16225,23130, 29770. | 51.0 (51.48) | 3.92 (4.51) | 13.72 (13.96) |
| 9 | $[Ni(L_1)_2(SO_4)]$ $C_{22}H_{18}NiN_6O_8S$ | 48 | 208-210 | 3.20 | 3280(NH),1635(C=O), 1612(C=N),525(M-N), 445(M-O) | 10265,19050, 26670. | 45.16 (45.55) | 3.07 (2.86) | 14.35 (14.18) |
| 10 | $[Ni(L_1)_2(NO_3)_2]$ $C_{22}H_{18}NiN_8O_{10}$ | 50 | 203-205 | 3.46 | 3280(NH),1642(C=O), 1610(C=N),515(M-N), 450(M-O). | 10580,18165, 27692. | 43.10 (43.41) | 2.93 (3.11) | 18.27 (18.33) |
| 11 | $[Ni(L_1)_2(C_2O_4)$ $C_{24}H_{22}NiN_6O_8$ | 48 | 199-201 | 3.33 | 3280(NH),1655(C=O), 1612(C=N),570(M-N), 445(M-O). | 11530,19045, 28115. | 49.61 (49.93) | 3.78 (4.09) | 14.45 (14.66) |
| 12 | $[Ni(L_1)_2(CH_3CO_2)_2$ $C_{26}H_{24}NiN_6O_8$ | 51 | 210-212 | 3.50 | 3280(NH),1675(C=O), 1615(C=N),510(M-N), 450(M-O). | 11285,18280, 27111. | 51.44 (51.51) | 3.95 (4.21) | 13.83 (14.13) |
| 13 | $[Co(L_2)_2(SO_4)]$ $C_{22}H_{18}CoN_6O_6S_3$ | 52 | 201-203 | 4.92 | 3280(NH),1633(C=O), 1612(C=N),525(M-N), 445(M-O),375(M-S). | 8575,17545, 22190. | 42.80 (43.26) | 2.91 (3.11) | 13.60 (13.28) |
| 14 | $[Co(L_2)_2(NO_3)_2]$ $C_{22}H_{18}CoN_8O_8S_2$ | 51 | 194-196 | 4.82 | 3280(NH),1642(C=O), 1614(C=N),515(M-N), 450(M-O),375(M-S). | 9170,18715, 21980. | 40.94 (41.33) | 2.78 (2.59) | 17.35 (17.33) |
| 15 | $[Co(L_2)_2(C_8O_4)]$ $C_{22}H_{18}CoN_6O_6S_2$ | 51 | 203-205 | 4.97 | 3280(NH),1652(C=O), 1610(C=N),515(M-N), 442(M-O),380(M-S). | 10215,17890, 22262. | 47.30 (47.01) | 2.95 (3.34) | 13.78 (13.91) |
| 16 | $[Co(L_2)_2(CH_3CO_2)]$ $C_{26}H_{24}CoN_6O_6S_2$ | 50 | 191-193 | 5.16 | 3280(NH),1648(C=O), 1615(C=N),525(M-N), 450(M-O),380(M-S). | 8140,17645, 23450. | 48.84 (42.88) | 3.75 (3.93) | 13.13 (12.94) |

| | | | | | | | | | |
|---|---|---|---|---|---|---|---|---|---|
| 17 | $[Cu(L_2)_2(SO_4)]$<br>$C_{22}H_{18}CuN_6O_6S_3$ | 50 | 210-212 | 1.55 | 3280(NH),1654(C=O),<br>1612(C=N),515(M-N),<br>445(M-O),380(M-S). | 16780,23585,<br>29415. | 42.48<br>(42.65) | 2.89<br>(3.11) | 13.50<br>(13.47) |
| 18 | $[Cu(L_2)(NO_3)_2]$<br>$C_{22}H_{18}CuN_8O_8S_2$ | 49 | 201-203 | 1.52 | 3280(NH),1651(C=O),<br>1610(C=N),525(M-N),<br>445(M-O),375(M-S). | 17555,23510,<br>30515. | 40.65<br>(40.77) | 2.76<br>(3.24) | 17.23<br>(17.01) |
| 19 | $[Cu(L_2)_2(C_2O_4)]$<br>$C_{24}H_{18}CuN_6O_6S_2$ | 48 | 194-197 | 1.57 | 3280(NH),1635(C=O),<br>1614(C=N),525(M-N),<br>450(M-O),382(M-S). | 16490,23135,<br>29715. | 46.95<br>(46.52) | 2.93<br>(3.36) | 13.68<br>(13.77) |
| 20 | $[Cu(L_2)_2(CH_3CO_2)_2]$<br>$C_{26}H_{24}CuN_6O_6S_2$ | 50 | 208-210 | 1.54 | 3280(NH),1643(C=O),<br>1612(C=N),515(M-N),<br>450(M-O),375(M-S). | 16225,22255,<br>29415. | 48.49<br>(48.67) | 3.72<br>(3.91) | 13.04<br>(12.84) |
| 21 | $[Ni(L_2)_2(SO_4)]$<br>$C_{22}H_{18}NiN_6O_6S_3$ | 49 | 199-201 | 3.45 | 3280(NH),1639(C=O),<br>1610(C=N),525(M-N),<br>445(M-O),380(M-S). | 10265,18285,<br>26670. | 42.81<br>(42.61) | 2.91<br>(3.38) | 13.61<br>(13.77) |
| 22 | $[Ni(L_2)_2(NO_3)_2]$<br>$C_{22}H_{18}NiN_8O_8S_2$ | 50 | 191-193 | 3.41 | 3280(NH),1645(C=O),<br>1614(C=N),510(M-N),<br>450(M-O),375(M-S). | 11530,19050,<br>29955. | 40.96<br>(41.44) | 2.79<br>(2.68) | 17.36<br>(17.65) |
| 23 | $[Ni(L_2)_2(C_2O_4)]$<br>$C_{24}H_{18}NiN_6O_6S_2$ | 51 | 201-203 | 3.34 | 3280(NH),1635(C=O),<br>1612(C=N),515(M-N),<br>445(M-O),382(M-S). | 10880,18610,<br>28555. | 47.32<br>(47.51) | 2.95<br>(3.26) | 13.79<br>(13.68) |
| 24 | $[Ni(L_2)_2(CH_3CO_2)_2]$<br>$C_{26}H_{24}NiN_6O_6S_2$ | 48 | 210-212 | 3.48 | 3280(NH),1652(C=O),<br>1614(C=N),525(M-N),<br>450(M-O),375(M-S). | 11245,19015,<br>26760. | 48.86<br>(48.90) | 3.75<br>(3.89) | 13.14<br>(12.95) |
| 25 | $[Co(L_3)_2(SO_4)]$<br>$C_{22}H_{20}CoN_8O_6S$ | 52 | 190-192 | 4.88 | 3280(NH),1644(C=O),<br>1612(C=N),510(M-N),<br>445(M-O). | 8140,17545,<br>21980. | 45.30<br>(45.78) | 3.42<br>(3.51) | 19.20<br>(19.06) |
| 26 | $[Co(L_3)_2(NO_3)_2]$<br>$C_{22}H_{20}CoN_{10}O_8$ | 50 | 198-200 | 5.20 | 3280(NH),1661(C=O),<br>1610(C=N),515(M-N),<br>450(M-O). | 10215,18770,<br>23450. | 43.23<br>(43.55) | 3.27<br>(3.11) | 22.90<br>(23.26) |
| 27 | $[Co(L_3)_2(C_2O_4)]$<br>$C_{24}H_{20}CoN_8O_6$ | 51 | 201-203 | 4.92 | 3280(NH),1650(C=O),<br>1610(C=N),515(M-N),<br>450(M-O). | 9415,17975,<br>22580. | 50.11<br>(50.37) | 3.47<br>(3.16) | 19.47<br>(19.58) |
| 28 | $[Co(L_3)_2(CH_3CO_2)]$<br>$C_{26}H_{26}CoN_8O_6$ | 52 | 195-197 | 4.86 | 3280(NH),1637(C=O),<br>1615(C=N),510(M-N),<br>445(M-O). | 10110,18222,<br>23145. | 51.59<br>(51.77) | 4.29<br>(4.10) | 18.50<br>(18.98) |
| 29 | $[Cu(L_3)_2(SO_4)]$<br>$C_{22}H_{20}CuN_8O_6S$ | 49 | 204-206 | 1.57 | 3280(NH),1643(C=O),<br>1610(C=N),510(M-N),<br>445(M-O). | 17465,22670,<br>30550. | 44.94<br>(45.38) | 3.40<br>(3.62) | 19.05<br>(18.77) |
| 30 | $[Cu(L_3)_2(NO_3)_2]$<br>$C_{22}H_{20}CuN_{10}O_8$ | 50 | 210-212 | 1.55 | 3280(NH),1665(C=O),<br>1612(C=N),510(M-N),<br>450(M-O). | 18250,23575,<br>29415. | 42.90<br>(43.21) | 3.24<br>(3.10) | 22.73<br>(22.89) |
| 31 | $[Cu(L_3)_2(C_2O_4)]$<br>$C_{24}H_{20}CuN_8O_6$ | 48 | 198-201 | 1.52 | 3280(NH),1638(C=O),<br>1614(C=N),525(M-N)<br>450(M-O). | 16915,22810,<br>30650. | 49.71<br>(49.38) | 3.44<br>(3.62) | 19.31<br>(19.47) |
| 32 | $[Cu(L_3)_2(CH_3CO_2)_2]$<br>$C_{26}H_{26}CuN_8O_6$ | 50 | 207-209 | 1.54 | 3280(NH),1641(C=O),<br>1612(C=N),515(M-N),<br>450(M-O). | 17580,22155,<br>29715. | 51.20<br>(51.55) | 4.26<br>(4.11) | 18.36<br>(18.53) |
| 33 | $[Ni(L_3)_2(SO_4)]$<br>$C_{22}H_{20}NiN_8O_6S$ | 52 | 211-215 | 3.34 | 3280(NH),1665(C=O),<br>1610(C=N),515(M-N),<br>450(M-O). | 10715,18280,<br>27730. | 45.32<br>(45.49) | 3.43<br>(2.95) | 19.21<br>(19.55) |
| 34 | $[Ni(L_3)_2(NO_3)]$<br>$C_{22}H_{20}NiN_{10}O_8$ | 50 | 202-205 | 3.49 | 3280(NH),1648(C=O),<br>1612(C=N),525(M-N),<br>445(M-O). | 10265,19050,<br>26670. | 43.25<br>(43.63) | 3.27<br>(3.14) | 22.91<br>(23.08) |
| 35 | $[Ni(L_3)_2(C_2O_4)]$<br>$C_{24}H_{20}NiN_8O_6$ | 51 | 198-200 | 3.38 | 3280(NH),1640(C=O),<br>1614(C=N),515(M-N),<br>445(M-O). | 11530,18285,<br>29955. | 50.13<br>(50.48) | 3.47<br>(3.52) | 19.48<br>(19.11) |
| 36 | $[Ni(L_3)_2(CH_3CO_2)_2]$<br>$C_{26}H_{26}NiN_8O_6$ | 50 | 208-211 | 3.41 | 3280(NH),1663(C=O),<br>1612(C=N),510(M-N),<br>450(M-O). | 10585,19045,<br>28190. | 47.44<br>(47.61) | 4.66<br>(4.84) | 20.11<br>(19.96) |

The solid state octahedral Co(II) complexes show $\mu_{eff}$ values between 4.85-5.22 B.M, corresponding to a $d^7$ electronic configuration[37]. Similarly, Ni(II) complexes show $\mu_{eff}$ values between 3.21-3.50 B.M respectively, matching fairly well with those observed for octahedral environment[38]. The magnetic susceptibility measurements for Cu(II) complexes show one unpaired electron per Cu(II) ion having $\mu_{eff}$ values 1.52-1.58 B.M showing distorted octahedral configuration[39,40] for Cu(II) complexes.

### Antibacterial Properties

Metal complexes of Schiff-base ligands lend themselves ideally to the studies of the effect of metal ions or anions. Our previous studies[16-27] in this regard give a detailed and systematic description enlightening this effect of metal ions (cations) on the pharmacological behaviour of biologically active compounds/antibacterial agent. However, in the present studies, elaboration of the role of anions (counter part of cations) in increasing the antibacterial activity in metal chelates is determined. Table 3 describes the results of these studies and it is interesting to note that when the same metal chelate/complex having different anions such as sulphate, nitrate, oxalate, and acetate was individually subjected to the evaluation of antibacterial activity, its degree of potency varied. For example, Co(II) complexes having nitrate anions as a counter part, were found to be more bactericidal than the Co(II) complexes having anions other than nitrate. Similarly, the same Co(II) complexes with oxalate anions were observed to be more antibacterial than the Co(II) complexes having acetate and sulphate anions. The identical results were found in Ni(II) and Cu(II) complexes.

**Table 3. Antibacterial Activity Data**

| Ligands/ Complexes | Microbial Species a | b | c |
|---|---|---|---|
| $L_1$ | - | + | ++ |
| $L_3$ | - | + | ++ |
| 1 | + | ++ | + |
| 2 | ++ | +++ | ++++ |
| 3 | ++ | ++ | +++ |
| 4 | + | ++ | +++ |
| 5 | + | ++ | ++ |
| 6 | ++ | +++ | +++ |
| 7 | +++ | ++ | +++ |
| 8 | ++ | ++ | +++ |
| 9 | ++ | ++ | + |
| 10 | ++ | ++++ | +++ |
| 11 | ++ | +++ | +++ |
| 12 | + | +++ | +++ |
| 13 | + | ++ | +++ |
| 14 | ++ | +++ | ++++ |
| 15 | ++ | +++ | +++ |
| 16 | ++ | +++ | ++ |
| 17 | + | ++ | ++ |
| 18 | +++ | +++ | + |
| 19 | ++ | +++ | +++ |
| 20 | + | ++ | ++++ |
| 21 | + | - | + |
| 22 | ++ | +++ | +++ |
| 23 | + | +++ | ++ |
| 24 | + | - | +++ |
| 25 | + | +++ | +++ |
| 26 | ++ | ++++ | ++++ |
| 27 | + | ++ | +++ |
| 28 | + | +++ | +++ |
| 29 | + | ++ | ++ |
| 30 | ++ | +++ | ++++ |
| 31 | + | +++ | ++ |
| 32 | + | ++ | +++ |
| 33 | + | ++ | ++ |
| 34 | ++ | ++++ | +++ |
| 35 | + | +++ | +++ |
| 36 | + | ++ | +++ |

a = Escherichia coli, b = Pseudomonas aeruginosa, c = Staphylococcus aureus
Inhibition zone diameter (mm), +: 6-10; ++: 10-14; +++: 14-18; ++++: 18-22.

From the obtained data, it was generally observed that the order of potency in comparison to the metal complexes having chloride anions reported earlier[27] and to the present studies having anions other than chloride against the tested bacterial species was found to follow the order as nitrate > oxalate > acetate > chloride > sulphate

In the light of the above observations, it is found that the role of anions in increasing the antibacterial activity of metal complexes is definite.

### ACKNOWLEDGEMENT

The sincere help of Department of Pathology, Qaid-e-Azam Medical College, Bahawalpur, in undertaking the antibacterial studies is gratefully acknowledged.

### REFERENCES

1 S. Kirschener, Y. K. Wei, D. Francis and J. Berjman, *J. Med. Chem.*, 1966, **9**: 369

2 A. Furst, "*Metal Binding in Medicine*", Lippincott Co, Philadelphia PA, 1960

3 W. E. C. Wacker and B. L. Vallee, *J. Biochem.*, 1959 **234**: 3257

4 R. J. P. William, *Quart. Rev. Chem. Soc.*, 1970, **24**: 331

5 H. Sigel and D. B. McCormick, *Accounts. Chem. Res.*, 1970, **3**: 201

6 D. R. Williams, *Chem. Rev.*, 1972, **72**: 203

7 R. S. Sirivastava, *Ind. J. Chem.*, 1990, **29**: 1024

8 L. Albanus, N. E. Bjorklurd, B. Gustafsson and M. Johsson, *Acta Pharmacol. Toxicol. Suppl.*, 1975, **36**: 93

9 I. Belsky, D. Gertner and A. Zilkha, *J. Med. Chem.*, 1968, **11**: 92

10 E. G. Sender, L. D. Wright and D. B. McCormick, *J. Biol. Chem.*, 1965, **240**: 3628

11 H. R. Mahler and E. H. Cordes, "*Biological Chemistry*", Harper and Rowe, New York, NY, 1966

12 M. V. Artmenko, E. A. Chityakova, K. E. Siyusarenko and O. B. Pritula, *Ukr. Khim. Zh.*, 1972, **38**: 137, *Chem. Abstr*, 1972, **76**: 120979s

13 N. Noyamalpass and V. Isikday, *Doga Bilim. Derg. Seri.*, 1985, **9**: 183, *Chem Abstr*, 1986, **103**: 210881j

14 M. Dixon and E. C. Webb, "*Enzymes*", 1964, Green and Co, London

15 B. L. Vallee and J. E. Coleman, *Compr. Biochem.*, 1964, **12**: 165

16 Z. H. Chohan and M. A. Farooq, *Pak. J. Pharmaceut. Sci.*, 1994, **7 (2)**: 45

17 Z. H. Chohan, *Chem. Pharm. Bull.*, 1991, **39 (6)**: 1578

18 Z. H. Chohan and H. Pervez, *Synth. React. Inorg. Met.- Org. Chem.*, 1993, **23(7)**: 1061

19 Z. H. Chohan and A. Rauf, *Pak. J. Med. Res.*, 1990, **29 (9)**: 207

20 Z. H. Chohan and A. Rauf, *J. Inorg. Biochem.*, 1992, **46**: 41

21 Z. H. Chohan and M. A. Farooq, *J. Chem. Soc. Pak.*, 1995, **17(1)**: 14

22 Z. H. Chohan and A. Rauf, *Pak. J. Med. Res.*, 1990, **29 (4)**: 210

23 Z. H. Chohan and A. Khaliq, *Pak. J. Med. Res.*, 1989, **28 (2)**: 92

24 Z. H. Chohan and H. Pervez, *Sci. Int.*, 1992, **4 (4)**: 345

25 Z. H. Chohan and H. Pervez, *J. Natural. Sci. Math.*, 1992, **32 (2)**: 241

26 Z. H. Chohan and H. Pervez, *J. Res(Sci).*, 1992, **4 (1)**: 45

27 Z. H. Chohan and A. Rauf, *Synth. React. Inorg. Met.- Org. Chem*, 1996, **26 (4)**: 591

28 E. Merck, "*A Handbook of Culture Media*", 1982, Frankfurter Straße 250, D - 6100 Darmstadt, Germany

29 W. J. Geary, *Coord. Chem. Rev.*, 1971, **7**: 81

30 K. Nakamoto, "*Infrared and Raman Spectra of Inorganic and Coordination Compounds*", 1963, John. Wiley, New York, NY

31 B. B. Kanl and K. B. Pandeya, *J. Inorg. Nucl. Chem.*, 1977, **40**: 1035

32 M. P. Teotia, D. K. Rastogi and W. Malik, *Inorg. Chim. Acta.*, 1973, **7**: 339

33 A. B. P. Lever, "*Inorganic Electronic Spectroscopy*", 1984, Elsevier, NY, 2nd Ed.

34 A. D. Liehr, *J. Physc. Chem.*, 1967, **67**: 1314

35 R. C. Aggarwal and C. Vallabhanevc, *Trans. Met. Chem.*, 1978, **3**: 309

36 A. B. P. Lever and D. Ogden, *J. Chem. Soc (A)*, 1967, 2041

37 C. J. Balhausen, "*An Introduction to Ligand Fields*", 1962, McGraw Hill, NY

38 B. N. Figgis and J. Lewis, *J. Prog. Inorg. Chem.*, 1967, **6**: 197

39 A. B. P. Lever, *Inorg. Chem.*, 1965, **4**: 763

40 M. D. Glick and R. L. Lintvedt, *Prog. Inorg. Chem.*, 1976, **21**: 233

**Received: September 10, 1996 - Accepted: September 17, 1996 - Received in revised camera-ready format: September 23, 1996**

